# Association of non-alcoholic fatty liver disease with left ventricular changes in treatment-naive patients with uncomplicated hypertension

**DOI:** 10.3389/fcvm.2022.1030968

**Published:** 2022-10-13

**Authors:** Cristiana Catena, Gabriele Brosolo, Andrea Da Porto, Debora Donnini, Luca Bulfone, Antonio Vacca, Giorgio Soardo, Leonardo A. Sechi

**Affiliations:** ^1^Internal Medicine and European Hypertension Excellence Center, Department of Medicine, University of Udine, Udine, Italy; ^2^Diabetes and Metabolism Unit, Department of Medicine, University of Udine, Udine, Italy; ^3^Liver Unit, Department of Medicine, University of Udine, Udine, Italy

**Keywords:** diastolic dysfunction, hypertrophy, insulin resistance, left ventricular mass, liver steatosis, remodeling

## Abstract

**Background and aims:**

Cardiac structural and functional changes have been demonstrated in patients with non-alcoholic fatty liver disease (NAFLD). Because of the frequent association of NAFLD with hypertension, we aimed to examine the relationship of liver steatosis with left ventricular (LV) changes in patients with hypertension.

**Materials and methods:**

In a cross-sectional study, we included 360 untreated, essential hypertensive patients who were free of major cardiovascular and renal complications. Liver steatosis was assessed by three different biochemical scores (NAFLD Liver Fat Score, LFS; Fatty Liver Index, FLI; Hepatic Steatosis Index, HSI). Echocardiography was performed with standard B-mode and tissue-Doppler imaging.

**Results:**

LV hypertrophy was present in 19.4% and LV diastolic dysfunction in 49.2% of patients who had significantly higher body mass index (BMI), blood pressure (BP), and homeostatic model assessment (HOMA) index and higher frequency of the metabolic syndrome and liver steatosis that was defined by presence of 2 or more positive scores. LV mass index increased progressively across patients who had none, 1, or 2 or more liver steatosis scores, with associated progressive worsening of LV diastolic function. LV mass index was significantly and positively correlated with age, BMI, BP, HOMA-index, LFS, and HSI. Logistic regression analysis showed that age, BP, and liver steatosis scores independently predicted LV hypertrophy and diastolic dysfunction. Liver steatosis independently predicted LV dysfunction but not LV hypertrophy even after inclusion in analysis of the HOMA-index.

**Conclusion:**

NAFLD is associated with LV hypertrophy and diastolic dysfunction in untreated patients with hypertension. In hypertension, NAFLD could contribute to LV diastolic dysfunction with mechanisms unrelated to insulin resistance.

## Introduction

Non-alcoholic fatty liver disease (NAFLD) is the most frequent chronic hepatic disease embracing a continuum of conditions progressing from plain liver steatosis to non-alcoholic steatohepatitis, cirrhosis and, eventually, hepatocellular carcinoma (1). NAFLD is commonly considered the distinctive feature of the liver involvement in the metabolic syndrome (2) and is an independent predictor of coronary heart and cerebrovascular disease (3). Moreover, NAFLD has been associated with subclinical cardiac and vascular changes that are known to precede major cardiovascular events (4). NAFLD is identified on liver biopsies when at least 5% of cells are repleted with fat in the presence of less than 30 g for men and 20 g for women of daily alcohol intake (5). Alternatively, hepatic imaging, elastography, or biochemical scores are used to detect liver steatosis without significant losses in specificity and sensitivity (6, 7). In recent years, the relationship of fatty liver with cardiac changes has been extensively investigated by use of either hepatic imaging or biochemical scores (8). Cross-sectional and prospective investigations conducted in the general population (9, 10) or selected groups of NAFLD patients (11–14), and cumulative analyses (15, 16) have reported significant association of fatty liver with left ventricular (LV) abnormalities. Moreover, recent studies demonstrate that fatty liver is associated with an increased risk of new onset arterial hypertension and, vice versa, that arterial hypertension is associated with an increased risk of NAFLD (17, 18), findings that indicate a close link between these two conditions.

Diastolic heart failure is detected with growing frequency in patients with hypertension and LV hypertrophy (19) and is preceded by impairment of LV filling properties that indicate reduced distensibility of the chamber. In hypertension, impaired LV diastolic properties can be found even in structurally normal LV and are associated with higher risk of death and hospitalization (20). Although response to an increased cardiac workload is crucial for the development of hypertension-related LV structural and functional changes, non-hemodynamic components might have a role. These components include aging, sex, body mass index, genetics, together with many biochemical, metabolic, and endocrine mediators (21). Therefore, in hypertension prompt detection of conditions that might contribute to LV hypertrophy and/or diastolic impairment is critical to forestall the decline of cardiac function and heart failure (22).

Although NAFLD, hypertension, and LV hypertrophy could share a common denominator in decreased insulin sensitivity, no information is currently available on the possible relevance of fatty liver for cardiac abnormalities in hypertensive patients. We hypothesized that NAFLD could contribute to subclinical cardiac changes of hypertensive patients and that this contribution occurs independently of insulin resistance. Therefore, this study was conducted to investigate the relationships between biochemical scores of liver steatosis and cardiac structural and functional abnormalities in untreated hypertensive patients who were free of cardiac, vascular, and renal complications.

## Materials and methods

### Design of the study and subjects

In a cross-sectional design, we enrolled never treated hypertensive subjects who were referred from July 2019 to December 2021 to our outpatient clinic. These patients include subjects with hypertension of all grades living in North-East of Italy and feature the hypertensive population of the geographical area (23). Blood pressure was measured with an automatic tool according to current guidelines (24). Measurements were done with an appropriately sized cuff after each subject had been supine for 15 min, and the average of three readings was recorded. Hypertension was diagnosed after repeated measurements that were obtained in 3 or more separate visits that were done at time intervals that varied according to the severity of hypertension (24). Predefined exclusion criteria were: age < 18 years or > 80 years; pregnancy or use of estrogens; body mass index (BMI) > 35 kg/m^2^; alcohol consumption > 30 g/day in men, and > 20 g/day in women; secondary hypertension; glomerular filtration rate < 30 ml/min/1.73 m^2^; history of recent illness; any type of liver disease including hepatotropic viral infections; any type of coronary artery, cerebrovascular, and peripheral vascular disease. Smoking was categorized when subjects had smoked for more than 5°years and up to 1°year before enrolment. Definition of ethanol intake was obtained by use of the AUDIT questionnaire (25). For 1°week, patients ate a standardized diet with a sodium content of approximately 150 mmol/day that was controlled by measurements of 24-h urinary sodium excretion. Causes of secondary hypertension were ruled out according to current guidelines (24) after thorough clinical, laboratory (urine analysis, glomerular filtration rate, plasma aldosterone, renin, and cortisol, free urinary cortisol and epinephrine, norepinephrine, and dopamine) and instrumental testing (electrocardiogram, cardiac ultrasound, renal ultrasound with assessment of the resistance indexes, and renal angio-computerized tomography and adrenal computerized tomography scan when indicated) (26).

An oral glucose tolerance test was performed in all patients with a standard load (75 g of glucose) and measurement of plasma glucose at 30, 60, 90, 120, and 180 min. According to current guidelines (27), patients were classified as having impaired fasting glucose (IFG) when fasting plasma glucose was between 100 and 125 mg/dL or glycated hemoglobin was between 5.7 and 6.5%, impaired glucose tolerance (IGT) when plasma glucose level at 120 min after oral glucose load was between 140 and 199 mg/dL, and diabetes when fasting plasma glucose was ≥ 126 mg/dL on at least two separate measurements, glycated hemoglobin ≥ 6.5%, or plasma glucose at 120 min after oral glucose load ≥ 200 mg/dL. None of diabetic patients was treated with insulin. Diagnosis of the metabolic syndrome was obtained by the American Heart Association criteria (28), when hypertension was associated with two or more of the following conditions: waist circumference ≥ 102 cm in men or ≥ 88 cm in women; fasting plasma glucose ≥ 100 mg/dL or use of hypoglycemic drugs; triglycerides ≥ 150 mg/dL; HDL-cholesterol ≤ 40 mg/dL in men or ≤ 50 mg/dL in women. The study followed the principles of the Declaration of Helsinki and received approval from the local Institutional Review Board. All patients provided their informed consent.

### Laboratory tests

Blood was collected by venipuncture into silicone-treated glass tubes after overnight fasting. After separation, plasma was stored at −80°C until assay. Measurements were done as previously reported (25). Glucose was measured by a chemical method, and insulin by radioimmunoassay. Insulin sensitivity was assessed by calculation of the homeostatic model assessment (HOMA) index using the formula {[fasting plasma glucose (mmol/L) × fasting insulin (μU/mL)/22.5]}. Total cholesterol, high-density lipoprotein, and triglycerides were measured in plasma in an automated device and low-density lipoprotein was calculated with the formula of Friedewald. Duplicate measurements of 24-h creatinine clearance that were normalized for body surface area were used to assess glomerular filtration rate. Plasma aldosterone was assayed by chemiluminescence (Immunodiagnostic System Ltd., London, England) and active renin by chemiluminescence enzyme immunoassay (CLIA).

### Liver steatosis

Liver steatosis was assessed by three different biochemical scores: NAFLD Liver Fat Score (LFS), Fatty Liver Index (FLI), and Hepatic Steatosis Index (HSI) (Table 1). These scores have been validated in population studies (6, 7). Liver steatosis was considered to be present when at least 2 of these scores indicated presence of fatty liver (LFS > 0.640; FLI > 60; and HIS > 36) (6, 7).

**TABLE 1:** Biochemical scores that were used for definition of liver steatosis.

Name of score	Variables included	Formula	Interpretation
NAFLD-LFS	Metabolic syndrome Type 2 diabetes Fasting serum insulin, mU/L Aspartate transaminase (AST), U/L Alanine transaminase (ALT), U/L	−2.89 + 1.18 x metabolic syndrome (yes: 1, no: 0) + 0.45 × type 2 diabetes (yes: 1, no: 0) × 0.15 × insulin in mU/L + 0.04 × AST in U/L −0.94 × AST/ALT	< 0.640 steatosis excluded > 0.640 indicates steatosis
FLI	Body mass index (BMI) Waist circumference, cm Serum triglycerides, mg/dL Serum gamma-glutamyl transpeptidase (GGT), U/L	[e^0.953*log^_*e*_^(triglycerides) + 0.139*BMI + 0.718*log^_*e*_^(GGT) + 0.053*waist circumference – 15.745^]/[1 + e^0.953*log^_*e*_^ (triglycerides) + 0.139*BMI + 0.718*log^_*e*_^ (GGT) + 0.053*waist circumference – 15.745^] × 100	< 30 steatosis excluded > 60 indicates steatosis
HIS	Gender Body mass index (BMI) Aspartate transaminase (AST), U/L Alanine transaminase (ALT), U/L Type 2 diabetes	8 × ALT/AST + BMI (+ 2 if type 2 diabetes present, + 2 if female)	< 30 steatosis excluded > 36 indicates steatosis

NAFLD-LFS, non-alcoholic fatty liver disease liver fat score; FLI, fatty liver index; HIS, hepatic steatosis index.

### Echocardiography

Echocardiography was performed with a standard machine (Aplio CV, Toshiba Medical System, Tokyo, Japan) by the same researcher who was blind to the patients’ characteristics (29, 30). LV end-systolic and end-diastolic diameters, left-atrial diameter, and thickness of the interventricular septum and LV posterior wall, and the relative wall thickness (RWT) were measured using a 2.5 MHz transducer, under bidimensional control. A RWT of 0.42 or more was considered to identify LV concentric geometry (31). The Penn Convention formula was used to calculate LV mass index, with a limit of 50 g/m^2.7^ for men and 47 g/m^2.7^ for women used to identify LV hypertrophy (31). LV ejection fraction and endocardial fractional shortening were measured to assess systolic function. Conventional pulsed Doppler measurement of early (E) and late-wave (A) trans-mitral peak velocities with calculation of the E/A ratio, and tissue-Doppler imaging (TDI) were used to assess LV diastolic function. TDI has higher sensitivity than conventional methodology for early detection of impaired LV diastolic function (32). Early (*e’*) and late (*a’*) myocardial velocities of the septal and lateral walls were measured in the mitral valve annulus, with a Doppler beam positioned parallel to the LV wall. Average *e’* and *a’* values were obtained from measurements in three consecutive cardiac cycles and the *e’/a’* and E/*e’* ratios were calculated. LV diastolic dysfunction was considered according to guidelines (32) when E/e’ ratio was > 14 or when 2 or more of the following variables were above or below a defined age-related cut-off: left atrial volume > 34 mL; E/A ratio 20–39 yr. < 1.1, 40–49 yr. < 0.9, 50–59 yr. < 0.8, 60–69 yr. < 0.7, ≥ 70 yr. < 0.6; e’ velocity < 55 yr. < 10 cm/sec, 55–65 yr. < 9 cm/sec, > 65 yr. < 8 cm/sec (30).

### Statistical analysis

The Kolmogorov-Smirnov test was used to assess normality of distribution of the study variables. Variables with skewed distribution were log transformed. Mean ± standard deviation is used to present normally distributed variables and median and [interquartile ranges] to present skewed variables. Categorical data are presented as absolute number and percentage. Frequency distributions were compared by the Pearson’ s chi-square test. Comparisons between 2 independent groups were done by the Student’s *t* and the Mann-Whitney test. Analysis of variance adjusted for covariates and with Bonferroni correction was used to compare more than 2 groups. Linear regression analysis was used to examine the relationships between continuously distributed variables, and correlation was expressed by the Pearson’s correlation coefficient *r*. Multivariate logistic regression models were used including LV hypertrophy or LV diastolic dysfunction as the dependent variables, to define variables that were independently associated. In these models, we included variables that were associated with LV hypertrophy and diastolic dysfunction in univariate analysis. *P* values of less than 0.05 were considered to indicate statistical significance. All data analyses were performed using Stata 12.1 (StataCorp LP, College Station, TX, USA).

## Results

Three-hundred-sixty treatment-naïve, patients with essential hypertension (age 48 ± 14 yr.; 199 males, 161 females) were included in this study. Clinical, biochemical, and cardiac ultrasound parameters are shown in Table 2 in which patients are separated based upon the presence (*n* = 70, 19.4%) or absence (*n* = 290, 80.6%) of LV hypertrophy, and presence (*n* = 172, 47.8%) or absence (*n* = 188, 52.2%) of LV diastolic dysfunction. Patients with LV hypertrophy and diastolic dysfunction had significantly higher BMI, waist circumference, systolic and diastolic blood pressure, plasma insulin, and HOMA-index, whereas renal function, plasma lipid fractions, and liver enzymes were comparable with patients with normal LV mass and diastolic function. LV diastolic dysfunction was also associated with higher alcohol consumption. Higher frequency of concentric geometry and worse diastolic function were associated with LV hypertrophy, and greater LV mass was associated with LV diastolic dysfunction. LV systolic function was normal in all patients and this was irrespective of absence or presence of either LV hypertrophy or LV diastolic dysfunction.

**TABLE 2:** Clinical characteristics and biochemical and echocardiographic variables of hypertensive patients who were grouped according to presence or absence of left ventricular hypertrophy or according to presence or absence of left ventricular diastolic dysfunction.

Variables	Hypertensive patients			*P*-value	Hypertensive patients		*P*-value
	All patients (*n* = 360)	LV hypertrophy No (*n* = 290)	LV hypertrophy Yes (*n* = 70)		Diastolic dysfunction No (*n* = 188)	Diastolic dysfunction Yes (*n* = 172)	
***Clinical characteristics***							
Age, y	48 ± 14	48 ± 14	51 ± 14	0.109	47 ± 15	50 ± 12	0.038
Male sex, n (%)	199 (55)	159 (55)	40 (57)	0.727	99 (53)	100 (58)	0.296
Body mass index, kg/m^2^	27.2 ± 4.7	26.7 ± 4.7	29.1 ± 4.4	< 0.001	26.0 ± 4.4	28.4 ± 4.8	< 0.001
Waist/hip ratio	0.89 ± 0.10	0.89 ± 0.10	0.93 ± 0.09	0.002	0.88 ± 0.11	0.91 ± 0.09	0.005
Systolic blood pressure, mm Hg	147 ± 18	145 ± 17	157 ± 20	< 0.001	144 ± 17	151 ± 19	< 0.001
Diastolic blood pressure, mm Hg	92 ± 13	91 ± 12	96 ± 13	0.002	90 ± 13	95 ± 12	< 0.001
Smokers, n (%)	78 (22)	22 (23)	9 (22)	0.133	40 (21)	38 (22)	0.851
Alcohol intake, g/d	10 ± 8	11 ± 7	10 ± 8	0.298	9 ± 8	11 ± 7	0.012
IFG/IGT/T2DM, n (%)	106 (29)	76 (26)	30 (43)	0.006	44 (23)	62 (36)	0.009
Metabolic syndrome, n (%)	84 (23)	59 (20)	25 (36)	0.006	30 (16)	54 (31)	< 0.001
***Biochemical variables***							
Creatinine clearance, mL/min/1.73°m^2^	96 ± 27	96 ± 26	96 ± 30	1.000	98 ± 29	95 ± 24	0.288
24-h urinary sodium, mmol/d	147 ± 76	148 ± 76	143 ± 75	0.621	150 ± 85	143 ± 764	0.384
Glucose, mg/dL	91 ± 22	90 ± 20	93 ± 19	0.256	91 ± 22	91 ± 19	1.000
Insulin, mU/L	9.5 ± 7.9	8.6 ± 6.0	11.3 ± 12.3	0.008	8.7 ± 7.1	10.5 ± 8.8	0.033
HOMA-index	1.56 [1.01–2.37]	1.49 [1.03–2.34]	1.75 [1.01–2.69]	0.049	1.46 [0.91–2.28]	1.61 [1.15–2.50]	0.016
Triglycerides, mg/dL	116 ± 84	118 ± 111	124 ± 69	0.666	116 ± 106	121 ± 99	0.645
Cholesterol, mg/dL	205 ± 42	206 ± 42	203 ± 41	0.590	205 ± 40	206 ± 42	0.817
HDL-cholesterol, mg/dL	57 ± 18	58 ± 19	54 ± 16	0.105	58 ± 19	56 ± 17	0.295
LDL-cholesterol, mg/dL	124 ± 36	124 ± 35	123 ± 40	0.835	122 ± 35	127 ± 36	0.183
AST, U/L	22 ± 8	22 ± 7	23 ± 10	0.328	22 ± 7	23 ± 10	0.269
ALT, U/L	26 ± 17	25 ± 17	28 ± 17	0.186	24 ± 11	27 ± 21	0.087
GGT, U/L	34 ± 34	32 ± 30	38 ± 36	0.150	33 ± 28	35 ± 36	0.555
AP, U/L	69 ± 29	67 ± 22	72 ± 24	0.095	69 ± 22	68 ± 35	0.744
Albumin, g/dL	4.37 ± 0.47	4.40 ± 0.46	4.24 ± 0.49	0.064	4.40 ± 0.46	4.34 ± 0.48	0.227
Active renin, μUI/mL	9.2 [3.7–18.6]	9.9 [4.2–19.2]	5.1 [2.5–13.5]	0.202	9.5 [3.7–19.3]	9.2 [3.7–18.6]	0.776
Aldosterone, pg/ml	119 ± 84	118 ± 87	124 ± 75	0.596	115 ± 85	123 ± 83	0.368
***Echocardiographic variables***							
LV end-diastolic diameter, mm	49 ± 5	48 ± 5	51 ± 5	< 0.001	48 ± 6	49 ± 5	0.088
LV end-systolic diameter mm	29 ± 5	28 ± 5	31 ± 5	< 0.001	28 ± 5	30 ± 5	< 0.001
Interventricular septum, mm	9.6 ± 2.0	9.1 ± 1.6	11.8 ± 2.0	< 0.001	9.3 ± 1.9	9.8 ± 2.0	0.016
Posterior wall, mm	9.0 ± 1.9	8.5 ± 1.5	11.1 ± 1.8	< 0.001	8.7 ± 1.7	9.4 ± 1.9	< 0.001
LVmass, g	163 ± 53	146 ± 38	230 ± 54	< 0.001	153 ± 50	174 ± 54	< 0.001
LVmass index, g/m^2^^.^^7^	38.5 ± 11.3	34.1 ± 6.9	56.6 ± 7.4	< 0.001	36.1 ± 1.6	41.2 ± 11.4	< 0.001
Relative wall thickness,%	0.384 ± 0.080	0.368 ± 0.071	0.450 ± 0.083	< 0.001	0.377 ± 0.081	0.392 ± 0.078	0.075
LV ejection fraction,%	69 ± 7	69 ± 6	68 ± 8	0.244	69 ± 7	68 ± 7	0.177
LV fractional shortening,%	40.8 ± 6.8	41.2 ± 6.8	39.2 ± 6.4	0.056	41.2 ± 7.2	40.4 ± 6.3	0.265
Left atrial volume, mL	36 ± 6	35 ± 6	40 ± 8	< 0.001	35 ± 6	38 ± 7	< 0.001
E/A ratio	1.21 ± 0.47	1.25 ± 0.48	1.07 ± 0.42	0.004	1.42 ± 0.48	0.96 ± 0.30	< 0.001
*e’*velocity, cm/s	10.1 ± 3.0	10.5 ± 3.0	8.3 ± 2.6	< 0.001	12.4 ± 2.3	7.9 ± 1.6	< 0.001
*e’/a’* ratio	1.23 ± 0.53	1.24 ± 0.55	0.96 ± 0.31	< 0.001	1.50 ± 0.56	0.90 ± 0.26	< 0.001
E/*e’* ratio	10.6 ± 3.3	9.8 ± 3.0	13.9 ± 3.8	< 0.001	6.9 ± 2.5	14.6 ± 3.9	< 0.001
***Liver steatosis***							
Steatosis, n (%)	96 (27)	68 (23)	28 (40)	0.005	37 (20)	59 (34)	0.002

Values are expressed as mean ± SD. Median and interquartile range in square brackets are shown for variables with skewed distribution. Reported *P* values for the comparison between patients with or without left ventricular hypertrophy and with or without left ventricular diastolic dysfunction. IFG, impaired fasting glucose; IGT, impaired glucose tolerance; T2DM, type-2 diabetes mellitus; HOMA, homeostasis model assessment; HDL, high-density lipoprotein; LDL, low-density lipoprotein; AST, aspartate aminotransferase; ALT, alanine aminotransferase; GGT, gamma-glutamyl transferase; AP, alkaline phosphatase; LV, left ventricle; E, early wave transmitral diastolic velocity; A, late-wave transmitral diastolic velocity; *e*’, early diastolic velocity of septal and lateral myocardial portions at tissue-Doppler imaging; *e*’/*a*’, early:late diastolic velocity ratio at tissue-Doppler imaging; *E*’/*e*’, E-wave transmitral velocity to early diastolic velocity at tissue-Doppler imaging ratio. Steatosis was defined by presence of ≥ 2 positive liver steatosis scores.

Liver steatosis was as defined by presence of at least 2 positive NAFLD scores and was found in 96 (26.7%) of the study patients. Among the remaining patients, 159 (44.2%) had no positive NAFLD score and 105 (29.2%) had only one. Prevalence of steatosis was significantly higher in patients with LV hypertrophy than in patients with normal LV mass (40.0% vs. 23.4%; *P* = 0.005), and in patients with LV diastolic dysfunction than in patients with normal diastolic function (34.3% vs. 19.7%; *P* = 0.002) (Table 1).

**FIGURE 1:**
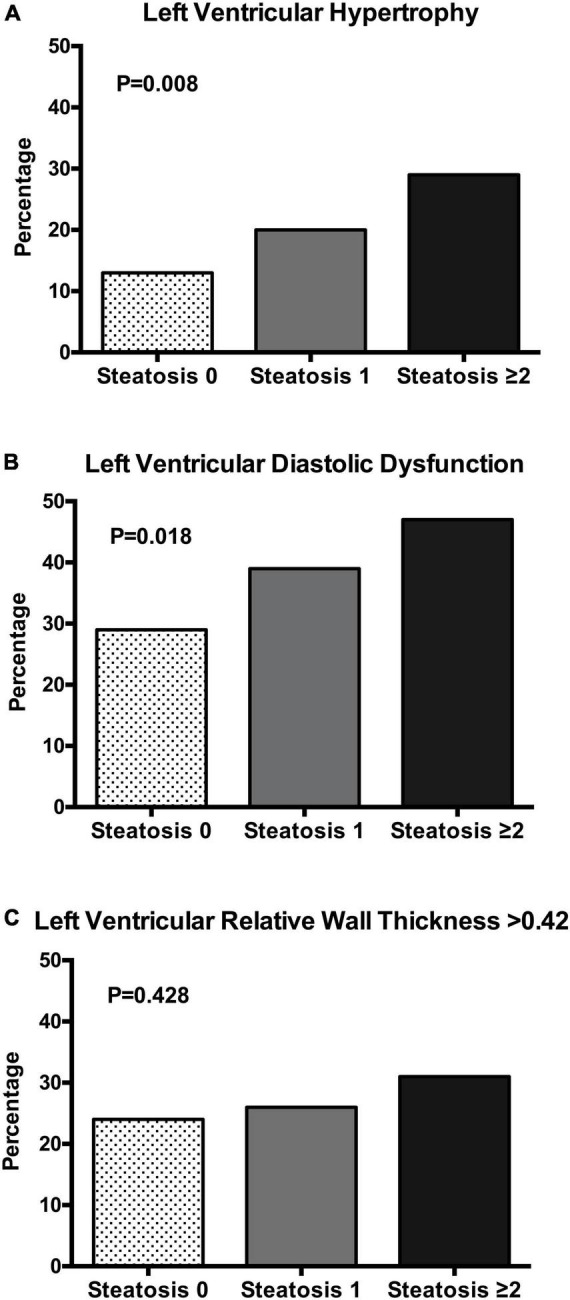
Bar graph showing the prevalence of left ventricular hypertrophy **(A)**, left ventricular diastolic dysfunction **(B)**, and left ventricular relative wall thickness **(C)** in patients with essential hypertension according to the number of liver steatosis scores (none, 1, ≥ 2). The chi square test was used to compare the frequency in the three groups.

Patients were further separated based upon presence of none, 1, or 2 or more positive NAFLD scores and their clinical, biochemical, and echocardiographic variables are shown in Table 3. Increasing number of NAFLD scores was associated with older age, male sex, and significantly higher BMI, waist/hip ratio, systolic and diastolic blood pressure, daily alcohol consumption, plasma insulin, HOMA-index, plasma lipids, and liver enzymes. No differences were found in glomerular filtration rate, and plasma renin and aldosterone levels. LV mass index increased progressively across liver steatosis groups and LV diastolic function (left atrial volume, E/A ratio, TDI *e’* velocity, and *e’/a’* ratio) progressively worsened. No differences were found in LV systolic function. Progressively higher frequency of LV hypertrophy and LV diastolic dysfunction (Figures 1A,B) was observed across liver steatosis groups, while no significant difference was observed in RWT (Figure 1C).

**TABLE 3:** Clinical characteristics and biochemical and echocardiographic variables of hypertensive patients who were grouped according to absence, or presence (1 or ≥ 2) of positive liver steatosis scores.

Variables	Hypertensive patients			*P*-value
	Steatosis 0 (*n* = 159)	Steatosis 1 (*n* = 105)	Steatosis ≥ 2 (*n* = 96)	
***Clinical characteristics***				
Age, y	48 ± 14	51 ± 14	46 ± 12	0.030
Male sex, n (%)	75 (47)	51 (49)	73 (76)	< 0.001
Body mass index, kg/m^2^	23.7 ± 2.5	28.5 ± 3.2	31.4 ± 4.7	< 0.001
Waist/hip ratio	0.85 ± 0.10	0.90 ± 0.09	0.97 ± 0.07	< 0.001
Systolic blood pressure, mm Hg	143 ± 18	148 ± 18	153 ± 18	< 0.001
Diastolic blood pressure, mm Hg	89 ± 13	94 ± 12	96 ± 10	< 0.001
Smokers, n (%)	29 (18)	20 (19)	29 (30)	0.059
Alcohol intake, g/d	9 ± 9	10 ± 9	13 ± 12	0.007
IFG/IGT/T2DM, n (%)	28 (18)	32 (30)	46 (48)	< 0.001
Metabolic syndrome, n (%)	4 (3)	17 (16)	63 (67)	< 0.001
***Biochemical variables***				
Creatinine clearance, mL/min/1.73°m^2^	97 ± 27	93 ± 25	98 ± 27	0.347
24-h urinary sodium, mmol/d	148 ± 74	144 ± 59	148 ± 89	0.898
Glucose, mg/dL	88 ± 17	91 ± 20	96 ± 18	0.003
Insulin, mU/L	5.9 ± 2.9	7.4 ± 3.9	14.7 ± 11.4	< 0.001
HOMA-index	1.19 [0.72–1.72]	1.42 [1.04–2.11]	2.52 [1.88–4.13]	< 0.001
Triglycerides, mg/dL	86 ± 38	112 ± 81	181 ± 158	< 0.001
Cholesterol, mg/dL	199 ± 41	211 ± 38	209 ± 45	0.033
HDL-cholesterol, mg/dL	63 ± 18	59 ± 18	45 ± 12	< 0.001
LDL-cholesterol, mg/dL	118 ± 37	127 ± 34	131 ± 37	0.014
AST, U/L	21 ± 7	21 ± 8	24 ± 10	0.009
ALT, U/L	19 ± 8	27 ± 16	36 ± 22	< 0.001
GGT, U/L	25 ± 21	33 ± 31	48 ± 48	< 0.001
AP, U/L	67 ± 22	71 ± 27	72 ± 40	0.343
Albumin, g/dL	4.35 ± 0.48	4.42 ± 0.45	4.35 ± 0.48	0.441
Active renin, μUI/mL	9.7 [3.9–19.9]	9.2 [3.7–18.1]	8.2 [3.1–15.5]	0.230
Aldosterone, pg/ml	113 ± 83	121 ± 80	126 ± 91	0.468
***Echocardiographic variables***				
LV end-diastolic diameter, mm	47 ± 5	49 ± 4	51 ± 5	< 0.001
LVend-systolic diameter mm	28 ± 5	29 ± 4	31 ± 5	< 0.001
Interventricular septum, mm	9.1 ± 1.9	9.8 ± 1.8	10.2 ± 2.1	< 0.001
Posterior wall, mm	8.6 ± 1.7	8.9 ± 1.7	9.8 ± 2.0	< 0.001
LVmass, g	144 ± 45	165 ± 44	191 ± 62	< 0.001
LVmass index, g/m^2^^.^^7^	34.9 ± 10.6	39.8 ± 9.7	43.0 ± 12.2	< 0.001
Relative wall thickness,%	0.376 ± 0.082	0.384 ± 0.074	0.398 ± 0.082	0.104
LV ejection fraction,%	69 ± 7	69 ± 6	68 ± 7	0.460
LV fractional shortening,%	41.4 ± 7.1	40.9 ± 6.3	39.8 ± 6.8	0.190
Left atrial volume, mL	34 ± 6	38 ± 5	39 ± 7	< 0.001
E/A ratio	1.28 ± 0.48	1.19 ± 0.48	1.14 ± 0.43	0.039
*e’* velocity, cm/s	10.7 ± 2.9	9.5 ± 2.9	9.7 ± 3.2	0.002
*e’/a’* ratio	1.29 ± 0.49	1.13 ± 0.43	1.08 ± 0.46	< 0.001
E/*e’* ratio	10.5 ± 3.5	10.6 ± 3.8	11.4 ± 4.1	0.156

Values are expressed as mean ± SD. Median and interquartile range in square brackets are shown for variables with skewed distribution. Reported *P* values for the comparison between patients with or without left ventricular hypertrophy and with or without left ventricular diastolic dysfunction. IFG, impaired fasting glucose; IGT, impaired glucose tolerance; T2DM, type-2 diabetes mellitus; HOMA, homeostasis model assessment; HDL, high-density lipoprotein; LDL, low-density lipoprotein; AST, aspartate aminotransferase; ALT, alanine aminotransferase; GGT, gamma-glutamyl transferase; AP, alkaline phosphatase; LV, left ventricle; E, early wave transmitral diastolic velocity; A, late-wave transmitral diastolic velocity; *e*’, early diastolic velocity of septal and lateral myocardial portions at tissue-Doppler imaging; *e*’/*a*’, early:late diastolic velocity ratio at tissue-Doppler imaging; *E*’/*e*’, E-wave transmitral velocity to early diastolic velocity at tissue-Doppler imaging ratio.

**FIGURE 2:**
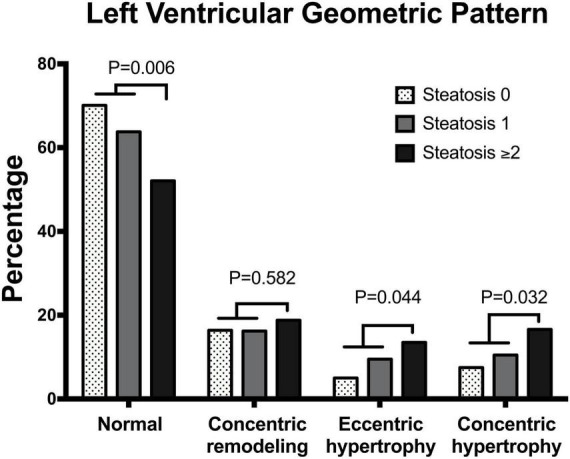
Bar graph showing the prevalence of different patterns of left ventricular geometry in patients with essential hypertension according to the number of liver steatosis scores (none, 1, ≥ 2).

Analysis of LV geometric patterns showed: normal geometry (*n* = 229, 63.6%), concentric remodeling (*n* = 61, 16.9%), eccentric hypertrophy (*n* = 31, 8.6%), and concentric hypertrophy (*n* = 39, 10.8%). Distribution of patterns of LV geometry among patients with different liver steatosis grading showed progressive reduction in the frequency of the normal pattern with significant increase of concentric and eccentric hypertrophy (Figure 2).

Analysis of univariate correlations showed significant an direct correlation of LV mass index with age, BMI, systolic and diastolic blood pressure, HOMA-index, and both LFS and HSI (Table 4). LFS and HIS were also significantly correlated with the left atrial volume. Relative wall thickness was correlated with blood pressure and LFS, while among LV diastolic variables, the E/A ratio and *e’* velocity was correlated directly with creatinine clearance and inversely with age and blood pressure.

**TABLE 4:** Univariate correlations with echocardiographic indexes as the dependent variables in hypertensive patients.

Variables	LV mass index		Relative wall thickness		Left atrial volume		E/A ratio		*e’*	
					
	*r*	*P*	*r*	*P*	*r*	*P*	*r*	*P*	*r*	*P*
Age	0.156	0.003	0.020	0.709	0.108	0.072	−0.563	< 0.001	−0.582	< 0.001
Body mass index	0.327	< 0.001	0.051	0.331	0.479	< 0.001	−0.097	0.096	−0.127	0.035
Systolic blood pressure	0.356	< 0.001	0.307	< 0.001	0.181	0.003	−0.153	0.009	−0.182	0.002
Diastolic blood pressure	0.249	< 0.001	0.240	< 0.001	0.127	0.035	−0.119	0.042	−0.133	0.029
Creatinine clearance	−0.007	0.892	−0.103	0.052	−0.094	0.120	0.280	< 0.001	0.156	0.003
HOMA-index	0.248	< 0.001	0.117	0.057	0.240	< 0.001	−0.030	0.661	−0.087	0.125
LFS	0.279	< 0.001	0.133	0.030	0.342	< 0.001	−0.033	0.633	−0.097	0.099
FLI	0.014	0.833	0.049	0.446	0.030	0.681	−0.036	0.622	−0.035	0.699
HSI	0.283	< 0.001	0.094	0.078	0.367	< 0.001	−0.096	0.100	−0.082	0.123

LV, left ventricle; E, early wave transmitral diastolic velocity; A, late-wave transmitral diastolic velocity; *e*’, early diastolic velocity of septal and lateral myocardial portions at tissue-Doppler imaging; LFS, liver fat score; FLI, fatty liver index; HIS, hepatic steatosis index. Scores of liver steatosis were treated as continuous variables.

Left ventricular (LV) hypertrophy was included as the dependent variable in a multivariate logistic model, and age, sex, systolic blood pressure, obesity, creatinine clearance, and liver steatosis as independent variables (Table 5). In this analysis, age, systolic blood pressure, and liver steatosis resulted independent predictors of LV hypertrophy. However, when the HOMA-index was added to the logistic model, the association of liver steatosis with LV hypertrophy failed (Table 6). In another model, LV diastolic dysfunction was included as the dependent variable with the same independent variables as in the previous model and the addition of LV hypertrophy (Table 7). In this model, age, systolic blood pressure, LV hypertrophy, and liver steatosis were independent predictors of LV diastolic dysfunction. Liver steatosis independently predicted LV diastolic dysfunction even after inclusion of the HOMA-index in the multivariate model (Table 8).

**TABLE 5:** Logistic regression analysis with left ventricular hypertrophy as the dependent variable.

Variable	Odds ratio	95% confidence interval	*P*-value
Age	1.02	1.00–1.05	0.035
Sex	0.85	0.46–1.58	0.609
Systolic blood pressure	1.03	1.01–1.05	< 0.001
Obesity	0.97	0.47–1.99	0.937
Creatinine clearance	1.00	0.99–1.01	0.460
Liver steatosis	1.53	1.05–2.21	0.025

Age, systolic blood pressure, duration of hypertension, and creatinine clearance were included in analysis as continuous variables. Sex (male/female), obesity (yes/no), and liver steatosis (none, 1, ≥ 2 positive liver steatosis scores) were included in analysis as categorical variables.

**TABLE 6:** Logistic regression analysis with left ventricular hypertrophy as the dependent variable and inclusion of the homeostatic model assessment (HOMA) index as an independent variable.

Variable	Odds ratio	95% confidence interval	*P*-value
Age	1.01	0.98–1.04	0.409
Sex	1.09	0.51–2.35	0.820
Systolic blood pressure	1.03	1.01–1.05	0.004
Obesity	1.34	0.58–3.11	0.489
Creatinine clearance	1.00	0.99–1.01	0.706
HOMA-index	1.00	0.99–1.01	0.117
Liver steatosis	1.46	0.93–2.31	0.103

Age, systolic blood pressure, duration of hypertension, creatinine clearance, and HOMA-index were included in analysis as continuous variables. Sex (male/female), obesity (yes/no), and liver steatosis (none, 1, ≥ 2 positive liver steatosis scores) were included in analysis as categorical variables.

**TABLE 7:** Logistic regression analysis with left ventricular diastolic dysfunction as the dependent variable.

Variable	Odds ratio	95% confidence interval	*P*-value
Age	1.03	1.00–1.05	0.002
Sex	1.29	0.79–2.10	0.302
Systolic blood pressure	1.01	1.00–1.03	0.028
Obesity	0.69	0.39–1.21	0.207
Creatinine clearance	0.99	0.99–1.00	0.258
Left ventricular hypertrophy	1.53	1.03–2.81	0.016
Liver steatosis	1.83	1.06–3.18	0.031

Age, systolic blood pressure, duration of hypertension, and creatinine clearance were included in analysis as continuous variables. Sex (male/female), obesity (yes/no), left ventricular hypertrophy (yes/no), and liver steatosis (none, 1, ≥ 2 positive liver steatosis scores) were included in analysis as categorical variables.

**TABLE 8:** Logistic regression analysis with left ventricular diastolic dysfunction as the dependent variable and inclusion of the homeostatic model assessment (HOMA) index as an independent variable.

Variable	Odds ratio	95% confidence interval	*P*-value
Age	1.03	1.01–1.06	0.004
Sex	1.48	0.83–2.63	0.184
Systolic blood pressure	1.02	1.01–1.05	0.019
Obesity	0.51	0.26–1.01	0.152
Creatinine clearance	1.00	0.98–1.01	0.513
HOMA-index	0.99	0.99–1.01	0.407
Liver steatosis	1.92	1.02–3.61	0.044

Age, systolic blood pressure, duration of hypertension, creatinine clearance, and HOMA-index were included in analysis as continuous variables. Sex (male/female), obesity (yes/no), left ventricular hypertrophy (yes/no), and liver steatosis (none, 1, ≥ 2 positive liver steatosis scores) were included in analysis as categorical variables.

## Discussion

A significant association of liver steatosis with LV abnormalities was reported in studies conducted in the general population and in subjects with NAFLD. Because LV changes have strong impact on the clinical outcome in hypertension, we examined the relationships of fatty liver with cardiac abnormalities in treatment-naïve, essential hypertensive patients who were free of major organ complications. Our results demonstrate that liver steatosis, as identified by use of biochemical scores, is significantly more frequent in hypertensive patients with LV hypertrophy and LV diastolic dysfunction than patients with normal hearts. LV mass index increases progressively and diastolic function worsens with increasing number of steatosis scores. Moreover, liver steatosis scores predict LV hypertrophy and diastolic dysfunction independently of blood pressure and obesity, although this association is independent of the HOMA-index only for LV diastolic dysfunction. This is the first demonstration that liver steatosis can contribute to LV changes in patients with essential hypertension. In these patients, mechanisms linking steatosis with LV diastolic dysfunction could be independent of insulin resistance.

The association of NAFLD with heart abnormalities has recently ended up in the spotlight because of the strong relevance that this relationship holds for the risk of cardiac failure and cardiovascular death. Cross-sectional investigations conducted with use of either liver biopsy or elastography in patients with NAFLD reported increased frequency of LV hypertrophy, concentric remodeling, and diastolic dysfunction (11–14). Similar findings were reported in a population-based study (33) and two meta-analyses of cross-sectional studies (15, 16). Later, significant association between NAFLD and LV hypertrophy was reported also in prospective investigations (8). In the CARDIA (Coronary Artery Risk Development in Young Adults) study, subjects with evidence of NAFLD at the 25th year of follow-up had higher rate of incident LV hypertrophy in the following 5 years (9). More recently, Li et al. have provided further prospective evidence of an association between the FLI and LV mass, replicating the findings obtained in two independent cohorts that included subjects of mixed ethnicity (10).

Although the epidemiologic link between hypertension and NAFLD has been clearly established (17, 18), information on the possible relationship of liver steatosis with hypertension-related cardiac changes is scanty. In 116 diabetic-hypertensive patients, NAFLD was associated with LV hypertrophy independently of BMI, systolic blood pressure, duration of diabetes, and glycated hemoglobin (34). Differently, in a preliminary study conducted in unselected patients with hypertension, patients with ultrasound evidence of NAFLD had comparable LV mass but lower E/A ratio on standard B-mode echocardiography than those without NAFLD, suggesting worse diastolic function (35). This study was performed in a significantly greater group of highly selected patients with hypertension who were free of important comorbidities and in whom LV function was assessed by TDI that is much more sensitive than conventional echocardiography in the early detection of impaired systolic and/or diastolic function. Also, because of the important cardiac specific effects of antihypertensive drugs, only treatment-naïve patients were included in the study. This constitutes one of the major strengths of the study, and strongly suggests that the association between fatty liver and both LV hypertrophy and diastolic dysfunction occurs independently of blood pressure levels and drug interference.

Nonalcoholic fatty liver disease (NAFLD) has received broad recognition as the hepatic manifestation of the metabolic syndrome, since insulin resistance and oxidative stress are essential requirements for fat accumulation in hepatocytes (36, 37). Insulin resistance has been closely linked also to hypertension (38) and LV changes (39), and abnormalities of insulin receptors were demonstrated both in NAFLD (40) and hypertension (41). In our hypertensive patients, higher plasma insulin and HOMA-index were associated with greater LV mass and diastolic dysfunction, and their values increased with increasing number of steatosis scores. These findings suggest that insulin resistance with the related hyperinsulinemia could have a leading role among the mechanism linking liver steatosis with hypertension-related LV changes. However, the association of liver steatosis with LV diastolic dysfunction remained independent after inclusion of the HOMA-index in the multivariate model, suggesting possible existence of additional mechanisms. Among these mechanisms, impaired iron metabolism and increased iron stores that are frequently observed in patients with NAFLD might activate oxidative stress and thereby unfavorably affect cardiovascular functions (42). Also, a prothrombotic state that is frequently detected in patients with fatty liver (43) might contribute to the development and progression of hypertension-related cardiac damage (44).

Limitations of the study need to be underlined. The cross-sectional design limits the possibility to establish a direct functional link between fatty liver and LV changes, although independence of reported associations would suggest so. Also, definition of liver steatosis by use of biochemical scores has intrinsic limitations, although these scores were validated with liver biopsies and are commonly used in studies with large numbers of patients. To maximize their predictive power, three separate scores were combined to define different levels of probability of liver steatosis.

## Conclusion

Characterization of conditions that in addition to an increased hemodynamic load contribute to cardiac abnormalities in hypertension is critical for the development of effective strategies to prevent or treat these abnormalities. Subclinical hypertension-related cardiac changes increase the risk of major cardiovascular events and anticipate progression to heart failure with preserved ejection fraction. Because of the previous evidence of an association between NAFLD and LV changes, we investigated the relevance of fatty liver in an accurately selected group of untreated hypertensive subjects. This is the first study to demonstrate an independent association of liver steatosis with LV hypertrophy and diastolic dysfunction in hypertension. This association can have great relevance in this specific group of patients. In addition to insulin resistance, additional mechanisms might have a role in this association. These findings open a window on the possibility to develop new strategies to prevent and treat hypertensive heart disease. Future studies will test the possible benefits of nutritional and pharmacologic interventions on liver steatosis for cardiac protection in hypertension.

## Data availability statement

The raw data supporting the conclusions of this article will be made available by the authors, upon reasonable request.

## Ethics statement

The studies involving human participants were reviewed and approved by the Internal Review Board. The patients/participants provided their written informed consent to participate in this study.

## Author contributions

CC, GB, GS, and LS: conceptualization and design. CC, GB, and LS: writing the original draft. AD, DD, LB, AV, and GS: writing—review and editing. All authors acquisition of data, data analysis, and approved the final version of the manuscript, including the authorship list and agree to be accountable for all aspects of the work in ensuring that questions related to the accuracy or integrity of any part of the work are appropriately investigated and resolved.
